# Vitamin D and Toxic Metals in Pregnancy - a Biological Perspective

**DOI:** 10.1007/s40471-024-00348-0

**Published:** 2024-06-20

**Authors:** Mandy Fisher, Hope A. Weiler, Jordan R. Kuiper, Michael Borghese, Jessie P. Buckley, Robin Shutt, Jillian Ashley-Martin, Anita Subramanian, Tye E. Arbuckle, Beth K. Potter, Julian Little, Anne-Sophie Morisset, Anne Marie Jukic

**Affiliations:** 1 Environmental Health Science and Research Bureau, Health Canada, Ottawa, ON Canada; 2 Nutrition Research Division, Health Products and Food Branch, Health Canada, Ottawa, ON Canada; 3 Milken Institute School of Public Health, The George Washington University, Washington, DC USA; 4 Department of Epidemiology, University of North Carolina at Chapel Hill, Gillings School of Global Public Health Sciences, Chapel Hill, North Carolina USA; 5 National Institute of Environmental Health Sciences (NIEHS), Duram, North Carolina USA; 6 School of Epidemiology and Public Health (SEPH), University of Ottawa, Ottawa, ON Canada; 7 School of Nutrition, Laval Université, Quebec, QC Canada

**Keywords:** Vitamin D, Metals, Pregnancy, Lead, Pb, Cadmium, Mercury, Arsenic

## Abstract

**Purpose of Review:**

To discuss the potential biological mechanisms between vitamin D and toxic metals and summarize epidemiological studies examining this association in pregnant women.

**Recent Findings:**

We identified four plausible mechanisms whereby vitamin D and toxic metals may interact: nephrotoxicity, intestinal absorption of metals, endocrine disruption, and oxidative stress. Few studies have examined the association between vitamin D and toxic metals in pregnant women. North American studies suggest that higher vitamin D status early in pregnancy are associated with lower blood metals later in pregnancy. However, a trial of vitamin D supplementation in a pregnant population, with higher metal exposures and lower overall nutritional status, does not corroborate these findings.

**Summary:**

Given ubiquitous exposure to many toxic metals, nutritional intervention could be a means for prevention of adverse outcomes. Future prospective studies are needed to establish a causal relationship and clarify the directionality of vitamin D and metals.

**Supplementary Information:**

The online version contains supplementary material available at 10.1007/s40471-024-00348-0.

## Introduction

Experts of nutritional sciences, medicine and environmental toxicology stress the growing need to further understand the interplay between environmental exposures, nutrition, and disease risk [1–7]. Toxic heavy metals are naturally occurring elements that have high atomic weight and density (≥ 5 times that of water) and include lead (Pb), mercury (Hg), cadmium (Cd), and arsenic [8]. Toxic metals are mainly non-biodegradable and tend to biomagnify in the food chain [9]. The main source of exposure for most people is food (e.g. arsenic in rice and shellfish, mercury in predatory fish) but other sources include water, soil, dust, and air (e.g., industrial and mining emissions, automobile exhaust, dental amalgam). In the case of Pb, common exposure sources include Pb-based paint chips, contaminated drinking water, some imported costume jewellery, and improperly glazed pottery or ceramic dishes [10–13]. Smoking [14, 15] and daily (or nearly daily) exposure to second-hand smoke [15] are prominent sources of Cd. A number of studies suggest that climate change may increase metals such as Hg in our food chain, especially in the North [16].

There are no known safe levels of exposure to toxic metals [17] and exposure is associated with a number of adverse biological effects including inflammation and oxidative stress [18, 19]. Exposure to toxic metals during pregnancy may be associated with the development of pregnancy complications (gestational diabetes, hypertensive disorders) as well as adverse birth outcomes including preterm birth and small for gestational age [20–23]. Moreover, these effects may persist well into later life, for both the gestational parent and child, potentially contributing to life-long risk of developing several chronic diseases (Type 2 Diabetes, hypertension). Dietary insufficiencies could further exacerbate the disease risk associated with metal exposures [1, 7]. In pregnancy, the vitamin D binding protein is reported to increase in order to meet the increased calcium demands and enhance tolerance to paternal and fetal alloantigens [24]. The biologically active form of vitamin D, 1,25-dihydroxyvtitamin D (1,25OHD), increases by two fold in 1st trimester and a further 2 to threefold throughout the course of the remainder of the pregnancy, and then rapidly declines at delivery [25]. Pregnancy therefore represents a critical point in time where the reduction of exposure to toxic metals, and the maintenance of adequate vitamin D levels, are paramount for both parent and child.

Vitamin D is a probable antioxidant [26] and may help to counteract the effects of toxic metals. The most widely accepted biomarker of vitamin D status is serum 25-hydroxyvitamin D (25OHD) (See eFigure 1)[27]. The Institute of Medicine guidelines suggest that 25OHD concentrations ≥ 50 nmol/L are sufficient for bone health in most people while serum concentrations > 125 nmol/L may be of concern [28]. Inadequate (defined as 25OHD levels below 40 nmol/L) or deficient (< 30 nmol/L) vitamin D status [28, 29] has been associated with higher risk of developing cardiovascular disease, poorer regulation of immune function, as well as menstrual cycle irregularities and adverse pregnancy outcomes such as preterm birth [30–32, 33•] However, key controversies exist in vitamin D research, including the definitions of insufficiency and inadequacy, and the role of free versus total 25OHD measurement in assessing adequacy [32, 34–39]. In North America the Recommended Dietary Allowance (RDA) of 600 IU for adults is set to ensure adequate vitamin D status for bone health; yet, there are no specific recommendations for vitamin D intake during pregnancy [40–42].

Identifying how environmental exposures such as toxic metals and nutrients relate during pregnancy is important for the health of both the offspring and of the mother over the long-term. In this narrative review, we have two objectives: 1) to discuss potential biological mechanisms underlying the associations between vitamin D and toxic metals and 2) to describe epidemiological studies that have examined the association between vitamin D metabolism and toxic metals in pregnant women.

## Methods

We conducted a narrative review to identify studies that examined vitamin D and metals (Pb, Hg, Cd or arsenic) using Health Canada librarian constructed search strategies in Medline, Embase, CAB Abstracts and Global Health on the Ovid platform, and in Scopus from database inception to January 19, 2024, limited to English language articles. A single author (MF) reviewed the citations yielded by the search to identify those that may be relevant to the review. Because this is a narrative and not a systematic review, we did not systematically track articles excluded at particular stages of the review.

We begin by providing an overview of vitamin D metabolism. For a more thorough review see Bikle 2014 [43]. We then describe mechanisms relevant to impacts of vitamin D and toxic metals. Finally, we review studies examining metals and vitamin D specifically in pregnant women.

### Vitamin D Metabolism

Vitamin D is a unique nutrient for two key reasons: 1) it is obtained both exogenously from diet and produced endogenously in the skin; 2) it is technically not a vitamin, but rather a “prohormone” that needs to be metabolically activated and converted to its hormonal form 1,25-dihydroxyvtitamin D (1,25OHD) through the vitamin D endocrine system (See eFigure 2–3). Vitamin D metabolism involves three main hydroxylation steps (25-hydroxylation, 1α-hydroxylation, and 24-hydroxylation) and all are performed by the cytochrome P450 mixed function oxidases (CYPs): CYP2R1, CYP27B1, and CYP24A1 [43]. Vitamin D_2_ and D_3_ obtained through the diet (food or supplements) are absorbed in the intestines and then delivered to various tissues through peripheral circulation via chylomicrons (triglyceride rich lipoproteins produced from dietary lipids), and subsequently transported to the liver through remnant lipoproteins. Endogenous production of vitamin D occurs during exposure of skin to UVB (spectrum 280–320 nm) rays from sunlight: 7-dehydrocholesterol (7-DHC) in the skin is converted to previtamin-D_3_, and then further converted to vitamin D_3_ by enthalpy [44].

The primary site of conversion of vitamin D to 25OHD from both vitamin D_2_ and D_3_ is in the liver [43]. The liver contains a number of CYPs with 25-hydroxylase activity, but CYP2R1 seems to be the major player in conversion of vitamin D to 25OHD [45]. 25OHD is transported from the liver to the kidneys via the vitamin D binding protein. In the kidneys, 25OHD is further hydroxylated (1α-hydroxylation) to 1,25OHD (calcitriol), which is the biologically active form of vitamin D. CYP27B1 is the major catalyst of 1,25OHD production, and mutations within this gene cause 1,25OHD deficiency, leading to rickets [46, 47]. CYP27B1 is tightly regulated [27, 43, 48, 49], and low calcium and elevated PTH stimulates CYP27B1 and the renal production of 1,25OHD. 1,25OHD itself limits CYP27B1 activity by inhibiting PTH and increasing fibroblast growth factor 23 (FGF23) production, a peptide synthesized in the bone, and that is essential to the maintenance of phosphate homeostasis by modulating intestinal phosphate absorption, renal phosphate reabsorption, and bone metabolism [50] (See eFigure 3). Usually the kidney is the major site of 1,25OHD synthesis, however PTH concentrations are reduced during pregnancy, suggesting that other tissues, cells or hormones may be involved in its production including the placenta [25, 51]. The placenta produces local 1,25OHD [52]. 25OHD crosses the placenta and 1,25OHD is produced in fetal kidneys. Cord blood 25OHD is about 50–80% of the concentration of maternal serum 25OHD [25].

The final step of vitamin D metabolism is the hydroxylation of vitamin D through the CYP24A1 enzyme, which has both 24- and 23-hydroxylase activity in humans (See eFigure 2). The main function of CYP24A1 is to prevent the accumulation of 25OHD and 1,25OHD, and subsequent toxicity [43].

### Potential Mechanisms for Biochemical Interactions between Vitamin D and Toxic Metals

**Fig. 1:**
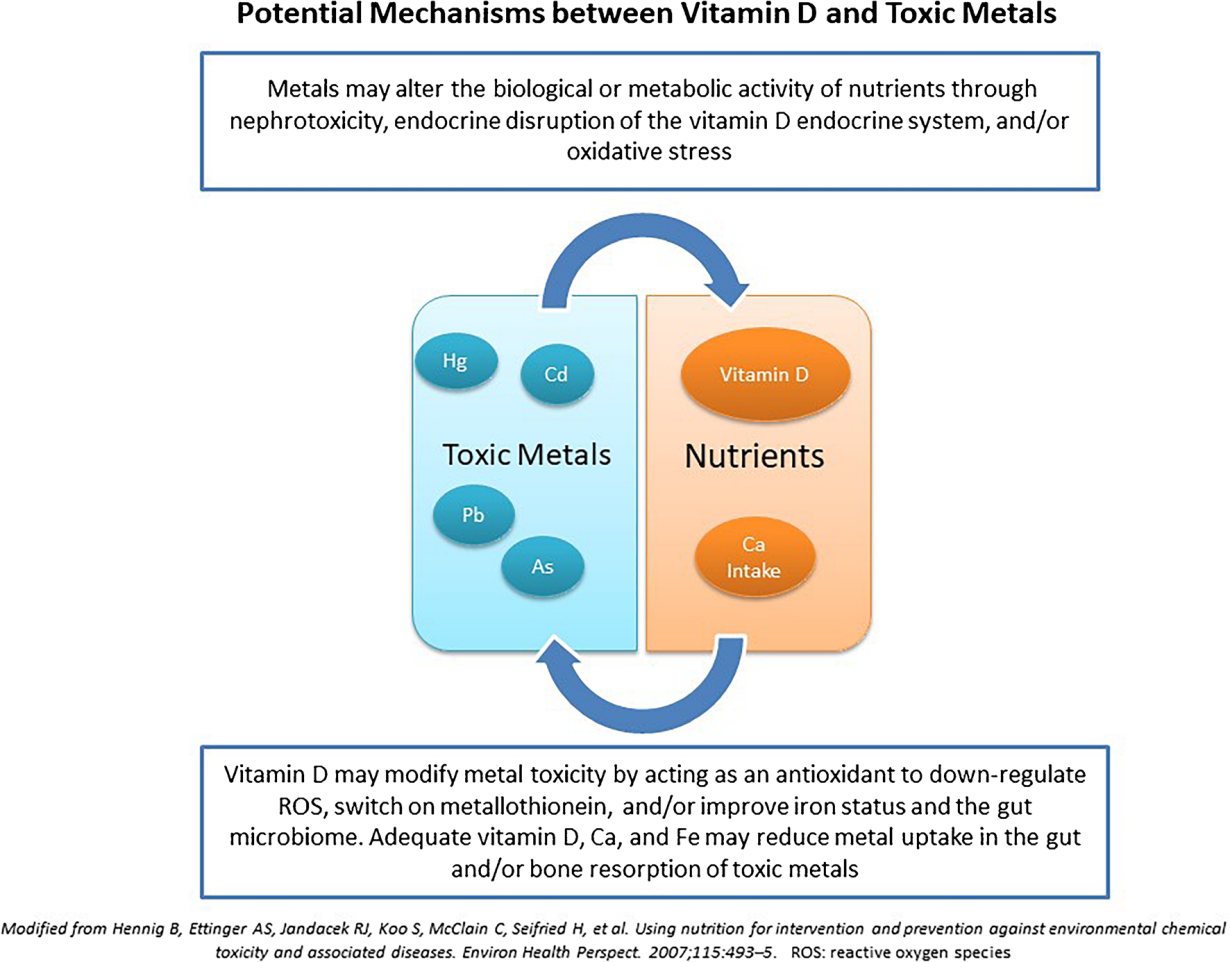
Potential mechanism between Vitamin D and Toxic Metals

There is a body of evidence indicating that long-term, high-level occupational or environmental contaminaton exposure to toxic metals is associated with lower vitamin D status [53–55]. However, less is known about lower doses of toxic metals that may also disrupt vitamin D metabolism through endocrine disruption, nephrotoxicity, and/or the inhibition of 1-α-hydroxylase enzyme in the renal tubules. In addition, although vitamin D is considered a probable antioxidant [26], metals have been shown to induce oxidative stress and reduce important antioxidants in the body, and this may in turn be associated with lower serum 25OHD [56]. Below is a discussion of these potential mechanisms whereby metals may disrupt vitamin D and where vitamin D may in turn modify the absorption or excretion of toxic metals (see Fig. 1).

#### Nephrotoxicity – Toxic Metals reducing Vitamin D Concentrations

High concentrations of toxic metals such as Pb and Cd are known to cause nephrotoxicity and affect vitamin D metabolism through the inhibition of CYP27B1 in the proximal renal tubules, therefore impairing renal hydroxylation of 25OHD to 1,25OHD [53–55, 57]. The resulting reduction in 1,25OHD in turn results in impaired calcium absorption across the intestinal tract and resorption or retention of Ca in the proximal renal tubules [53]. For example, the most severe form of chronic high dose Cd exposure is itai-itai disease, characterized by proximal tubular dysfunction in the kidneys and multiple bone fractures due to osteomalacia, identified in the Jinzu River basin of Toyama Prefecture, Japan [58]. The river had been heavily polluted with slag from an upstream mine, polluting the soil in rice paddies with high levels of Cd through irrigation. Patients exposed to Cd were found to have lower serum concentrations of the biologically active form of vitamin D, calcitriol (1,25OHD), and 24,25(OH)_2_D (calcitroic acid that can be excreted in urine) than non-exposed people [54, 59]. However 25OHD concentrations were similar across the Cd-exposed and non-exposed groups [54].

Several studies of occupational exposure to toxic metals have identified associations with 25OHD or 1,25OHD blood concentrations. For example two occupational studies in India, of battery manufacturer workers occupationally exposed to Pb described a negative correlation with serum 25OHD in exposed compared to age- and sex-matched unexposed group [60, 61]. However, few details were given in both studies on how the unexposed groups were selected. In Bangladesh, jewellery workers exposed to Pb fumes and dust for the last 10–25 years had, on average, significantly higher blood Pb levels (69 vs 15 µg/dL) and lower 25OHD concentrations (32 vs 81 nmol/L) compared to controls who were office workers not directly exposed to fumes but working in the same facility [53]. The authors mention that subjects and controls were age and environmental condition standardized. In a small (*n* = 19) cross-sectional study of male smelter workers from Israel, those with high Cd and low Pb showed the lowest concentrations of serum 1,25OHD, compared to those with either low or high levels of both contaminants [62]. There was no mention of control for smoking status and other potentially confounding factors in this study.

#### Intestinal Absorption of Toxic Cations – Low Vitamin D Status Increases Metal Absorption

Calcium and vitamin D play an important role in the intestinal absorption of metals. Blood Pb levels have been shown to increase during periods of high calcium demand [63–66] and this may be partly mitigated through calcium supplementation [67–69]. During pregnancy intestinal calcium absorption nearly doubles in order to meet the mineral demands of the fetus [70]. In states of adequate dietary intake of calcium and phosphate, these calcium ions are passively absorbed. However, when dietary intake is low (which is common for calcium in North America [71]), the parathyroid glands increase production of PTH, inducing the synthesis of 1,25OHD which in turn increases the intestinal lumen absorption of calcium. The synthesis of 1,25OHD depends on the availability of 25OHD. 1,25OHD binds with the VDR-retinoid X receptor (VDR-RXR) heterodimer to stimulate intestinal absorption of Ca. Intestinal absorption occurs through a transcellular pathway (apical calcium channel TRPV6), the calcium binding protein calbindin-D_9k_, and basolateral membrane calcium ATPaseD. Calbindin and other calcium binding proteins may be involved in intracellular transfer of calcium [47] and also bind other 2 + cations including Pb^2+^ and Cd^2+^ allowing for their absorption [55, 72]. However, experimental studies suggest that other mucosal proteins play an important role in the absorption of lead [73] (See eFigure 4).

Other evidence for intestinal mechanisms comes from the fact that female adults consistently show higher Cd levels than males in population based studies [15, 74, 75] which may be partially due to increased intestinal absorption of Cd due to generally lower iron levels [76]. Adequate vitamin D consumption may affect iron regulation and increase erythropoiesis in adults [77, 78].

Globally, the prevalence of anaemia is around 31% in females and 18% in males [79]. In pregnancy, iron needs are markedly increased to meet the demands of pregnancy (fetoplacental unit, expansion of the maternal erythrocyte mass) and to compensate for blood loss at delivery. In 80% of the world’s countries, anemia affects more than one-fifth of pregnancies [80]. One of the important mechanisms of iron absorption is through divalent metal transporter 1 (DMT1), which also absorbs other metal cations including Cd and Pb (with a higher affinity for Cd) [81, 82]. Several studies show inverse associations between blood ferritin with toxic metals (including Pb and Cd) in adults and children [83–85]. Lower preconception serum ferritin concentrations were associated with higher concentrations of Cd and Pb in the first trimester [86] while a longitudinal study observed that urinary Cd increased throughout pregnancy as iron stores were depleted [87]. In a national level population-based survey of Korean females over 10 years of age, lower serum 25OHD concentrations levels were associated with iron deficiency anemia and increased Cd concentrations in blood [82]. Vitamin D concentrations may affect hepcidin—an important regulator of iron metabolism [82, 88]. However, a vitamin D supplementation trial did not show an effect on serum hepcidin concentrations in a chronic disease population [89].

At a population level, for both children and adults, blood Pb levels have repeatedly been shown to follow a seasonal pattern among those living in temperate regions (e.g., USA, Canada, Europe), with higher concentrations in summer months relative to winter months [90, 91]. A reasonable explanation for this phenomenon is that cumulative sunlight exposure is greater in summer (warmer) months, thereby increasing vitamin D production and Ca^2+^-binding protein, subsequently increasing intestinal Ca (and by analogy, Pb) absorption [55]. However, it is also likely that exposure potential to Pb is highest in summer months, perhaps due to greater time spent outdoors interacting with contaminated soils (by which drier climates increase Pb-contaminated dust production), breathing in contaminated ambient air, and even a higher frequency of contaminated water intake. A prior study of Black and Hispanic children in Newark, NJ found that, as expected, children’s blood Pb levels were greater in summer months relative to winter months, for both children ages 1 to 3 years and 4 to 8 years [92]. However, seasonal differences in serum 25OHD concentrations were only observed for those in the ages 4 to 8 years group, with greater serum 25OHD concentrations observed in the summer (relative to winter) months and positively correlated with changes in blood Pb levels. This study demonstrates that the complex mechanisms of Pb (and likely other heavy metals) with vitamin D are likely age- and context-specific, and other mechanisms besides sunlight exposure inducing enhanced intestinal absorption of Pb must be at play.

We briefly mention two other downstream processes related to vitamin D and intestinal absorption of heavy metals. The first is the influence of inadequate Ca and vitamin D intake on bone demineralization. If intestinal absorption of calcium is insufficient, due to increased demand (as observed during pregnancy), Ca resorption from the bone to the blood stream occurs in order to maintain normal serum Ca levels. While this may result in both bone loss and limited bone mineralization [93], it also increases the likelihood of similarly shedding heavy metals stored in bone tissues back into the blood stream, thus “re-dosing” individuals [65]. The second is related to the gut microbiome, as there is some evidence to suggest that vitamin D may alter the composition of the gut microbiota [94], in turn decreasing absorption of toxic metals and potentially enhancing their elimination [95].

#### Endocrine Disruption – Toxic Metals Disrupt the Vitamin D Endocrine System

An endocrine disrupting chemical (EDC) is defined as a chemical or mixture of chemicals that interferes with normal hormone biosynthesis, signalling, or metabolism in the human body [96–99]. Although the kidney is the most well-known site of 25OHD hydroxylation to 1,25OHD, there are several other extra-renal tissues, including the placenta, bone, keratinocytes, and cells of the immune system (macrophages, T-lymphocytes, dendritic cells) that are also able to convert 25OHD to 1,25OHD [25, 51]. There are numerous tissues that express the VDR-RXR complex for the steroid hormone 1,25OHD suggesting that vitamin D may play a role in many other processes beyond calcium homeostasis [27]. Close to 200 genes are regulated by the VDR[100]. Therefore, most tissues that possess the enzyme 1α-hydroxylase could be considered endocrine tissues that convert 25OHD to the hormonal form 1,25OHD.

Toxic metals including Pb, Hg, Cd, and arsenic have established effects on the endocrine system and have altered physiological function in toxicological studies [98]. The hormonally active metabolite 1,25OHD is molecularly similar to other steroid hormones and similarly interacts with the VDR-RXR heterodimer [27, 101]. EDCs have the ability to act as ligands and attach to specific hormone receptors which bind to response elements in target genes, regulating gene expression – thus producing downstream effects (see eFigure 5)[9]. For example, Cd^2+^ has been observed to substitute for Zn^2+^ in the zinc finger DNA-binding domain of the estrogen receptors [9]. It has been demonstrated through high throughput screening that the VDR is activated or antagonised by a wide range of chemicals, including metals (Cd) [102]. If downstream effects are blocked or altered this could potentially increase the metabolism of vitamin D and reduce 25OHD. Other studies have observed toxic metals interfering with cytochrome P450 enzymes altering their expression and activity [103–105]. If metals alter the CYP enzymes involved in vitamin D metabolism, it could result in reduced synthesis of 25OHD and calcitriol.

Given this evidence, it seems plausible that endocrine disrupting effects of metals could alter the maintenance of the vitamin D endocrine system by interfering with any of its aspects including: induction or inhibition of the cytochrome P450 enzymes, vitamin D binding protein [106] or VDR-RXR heterodimer [107].

#### Oxidative Stress vs Antioxidant

##### Metals Induce Oxidative Stress to Disrupt Vitamin D Metabolism

The fourth potential mechanism associating vitamin D levels with metal exposure stems from the ability of metals to induce oxidative stress [108, 109] which may disrupt vitamin D metabolism. “Oxidative stress” is defined as an imbalance between cellular reactive oxygen species (ROS) and antioxidants [110]. ROS are “molecules containing at least one atom of oxygen and have the potential to generate free radicals”[111]. ROS are important for cellular function, however their excess can cause DNA, lipid, and protein damage. ROS are upregulated with infections as well as with Pb exposure [108, 112, 113].

Through immunostaining of in vitro placental tissue pieces, oxidative stress was shown to downregulate CYP2R1, as well as vitamin D binding protein, and the VDR, in preeclamptic verses normal pregnancies [114]. Increasing concentrations of blood Pb have been associated with decreased antioxidant activity in blood, including the antioxidant, glutathione peroxidase [56]. Dental workers exposed to Hg also show decreased antioxidant activity including glutathione peroxidase and superoxide dismutase [115, 116]. Animal studies show that antioxidants such as glutathione peroxidase improve 25OHD serum concentrations by upregulating genes involved in the expression of the vitamin D binding protein, CYP2R1, CYP27B1, and the vitamin D receptor. Glutathione peroxidase is positively associated with 25OHD serum concentrations in people who are obese and have type II diabetes [117, 118]. Supplementation with the antioxidant L-cysteine was shown to elevate glutathione, up-regulate 25-hydroxylase activity in the liver, and increase serum 25OHD concentration in obese adolescents [118].

##### Vitamin D Acts as Antioxidant to Reduce Metals

Conversely, micronutrients are known to down-regulate ROS [119, 120]. In vitro studies observed that vitamin D (1,25OHD) may play a role in switching on metallothioneins and their antioxidant properties [121, 122]. Metallothioneins are a super family of metal binding, low molecular weight, and cysteine rich proteins that are induced by (and bind to) a range of metal ions including arsenic, Cd, Hg and Pb [123]. Due to their metal binding properties, they are involved in the transport, storage, and detoxification of essential (Zn, Cu) and non-essential (As, Cd, Hg, Pb) metal ions [124–128]. Metallothioneins are also potent reactive oxygen scavengers and reduce lipid peroxidation by superoxide dependent hydroxyl radicals and hydrogen peroxide [124, 129]. In addition, metallothionein in human blood can be protective against cadmium toxicity, help with immune regulation, and prevent apoptosis [130]. Metallothionein gene expression is regulated by several factors including exposure to heavy metals [131]. For example, cadmium was reported to increase metallothionein gene expression and reduce the formation of sunburn cells [129], potentially through metallothionein’s antioxidant properties. Vitamin D might have a possible role in increasing metallothionein levels and promote the clearance of cadmium [121, 132]. It has been shown that 1,25(OH)D induces the expression of metallothionein mRNA in keratinocytes, liver, kidney and skin [121]. In turn, metallothionein can potentially induce the expression of 1,25OHD and act as an antioxidant [129].

Thus, vitamin D’s role as an antioxidant may help to downregulate the oxidative stress induced by metals at the time of exposure and prevent disruption to vitamin D homeostasis. However, if you are exposed to metals and have low vitamin D status, then metals may be more oxidizing and may exacerbate the effects of low vitamin D.

### Review of Pregnancy Studies

Six identified studies examined the association between vitamin D and metals during pregnancy (see Supplemental Table 1). A recent study from the U.S. LIFECODES pregnancy cohort (*n* = 381), reported that low concentrations of 25OHD (< 50 nmol/L) in the first trimester of pregnancy were associated with a 47% increase in 2nd trimester urinary Pb concentrations [133]. The Albany, New York Pregnancy Infancy Lead Study (APILS) [134], reported that a two standard deviation reduction in average vitamin D intake across pregnancy derived from a food questionnaire (10.5 to 2.4 mg) was associated with a 0.18 µg/dl increase in cord blood Pb concentrations. Similarly, a Canadian pregnancy cohort, observed that vitamin D intake in the highest quartile during the 1st trimester (ascertained via food frequency questionnaire) was associated with lower maternal 3rd trimester blood Cd (0.19 vs 0.21 µg/L), as well 1% lower cord blood Pb [135]. In this cohort a bidirectional analysis using cross-lagged panel models found that each log2 increase in 1st trimester 25OHD was associated with 9% (95% CI: -15%, -3%) lower 3rd trimester Cd and 3% (95% CI: -7%, 0.1%) lower Pb [136]. No association was observed between 1st trimester toxic metals (Cd and Pb) and 3rd trimester 25OHD, indicating that metals do not affect vitamin D levels. In a prospective pregnancy cohort from China, each doubling in urinary Cd averaged across pregnancy (1st, 2nd, 3rd trimester) was associated with 7% (95% CI: -13.9%, 1.0%) lower 25OHD concentrations in cord blood while arsenic was associated with 8% higher (95% CI: 0.8%, 16.3%) 25OHD concentrations [137]. Finally, in a secondary analysis of the Maternal Vitamin D for Infant Growth (MDIG) trial in Bangladesh [138••], pregnant participants in their 2nd trimester were randomized to receive weekly doses of 4,200, 16,800 or 28,000 IU of vitamin D_3_ throughout pregnancy. The results did not show any association between vitamin D_3_ dose with metal concentrations during pregnancy, but there was an increase in cord blood Pb and Cd concentrations among higher dosed groups compared to placebo.

In summary, of the few studies that have examined vitamin D and metals in pregnant women, observational studies in North America suggest that higher vitamin D intake or serum concentrations of 25OHD are associated with lower blood concentrations of some toxic metals during pregnancy; perhaps supporting the role of vitamin D as an antioxidant. In contrast, a supplementation trial among pregnant women with considerably higher metal (See Supplemental Table 1) and lower 25OHD concentrations suggests higher cord blood metals with vitamin D supplementation in pregnancy; potentially suggesting higher metal absorption through the gut with increased vitamin D intake. The differences across these studies could be due to the varying exposure levels, study design, and nutritional status (e.g. baseline 25OHD, anemia) of the participants. There may be unmeasured confounding, as 25OHD may be a marker of overall mineral status [139] or an indicator of physical activity, as seen in studies of adolescents and post-menopausal women [140, 141].

## Conclusions

Based on the available evidence to date, we propose at least four plausible mechanisms for the interaction of metals with vitamin D. There is evidence from the literature that long-term, high levels of exposure to metals, as observed in populations in the Jinzu River basin of Toyama Prefecture, Japan, can cause renal damage, and impact a person’s vitamin D status. However, the effect of toxic metals at lower doses and the direction of the association is unclear, and few studies have examined this association in pregnant women.

If a causal relationship is determined, improved vitamin D status could be a practical means for intervention and prevention of adverse outcomes associated with metal exposures, given the ubiquitous nature of many toxic metals [7, 142]. The prevalence of vitamin D deficiency (< 30 nmol/L) is estimated at 8.4% in Canada [143] and nearly one-fifth of Canadians (19%) and Americans (18%) have 25OHD concentrations < 40 nmol/L. In addition, young adults of reproductive age between 19–30 years (OR: 2.33, 95% CI: 1.66, 3.29) and 31–50 years (OR: 1.43, 95% CI: 1.03, 1.99) are more likely to have vitamin D concentrations < 40 nmol/L compared to older adults (71–79 year olds). Many countries report vitamin D deficiency (< 30 nmol/L) among > 20% of their population including India, Tunisia, Pakistan and Afghanistan [144]. It is also evident that metals exposure is a continuing problem. For example, lithium and cobalt production will likely undergo exponential growth over the coming decades due to the demand for lithium-ion batteries in electric vehicles [145]. Metals exposure is increasing through solar panel production (and disposal), which includes heavy metals like Pb [146], but also electronic waste, generally [147]. Moreover, climate change is projected to cause increased soil leaching and runoff of metals such as Cd [148]. Given this changing global context for metal exposures, there is a need for improving our understanding of the interactions between toxic metals and vitamin D with prospective studies that can clarify the causal pathways and directionality of the observed associations.

### Supplementary Information

Below is the link to the electronic supplementary material.

Supplementary file1 (PDF 24790 KB)
