# Supplementary material for: Vitamin D and Toxic Metals in Pregnancy - a Biological Perspective
Source: Curr Epidemiol Rep. 2024 Jun 20;11(3):153–63. doi: 10.1007/s40471-024-00348-0 (PMC11329583; doi:10.1007/s40471-024-00348-0)
Supplement: Supplementary file 1 — Supplementary file1 (PDF 24790 KB) [file 40471_2024_348_MOESM1_ESM.pdf]

Supplemental Information: Vitamin D and Toxic Metals in Pregnancy - a biological perspective

Mandy Fisher[1], Hope A. Weiler[2], Jordan R. Kuiper[3], Michael Borghese[1], Jessie P. Buckley[4], Robin Shutt[1], Jillian Ashley-Martin[1], Anita Subramanian[5], Tye E. Arbuckle[1], Beth K. Potter[6], Julian Little[6], Anne-Sophie Morisset[7], Anne Marie Jukic[5]

Author Affiliations

1. Environmental Health Science and Research Bureau, Health Canada; Ottawa, Ontario Canada;
2. Nutrition Research Division, Health Products and Food Branch, Health Canada; Ottawa, Ontario, Canada
3. Milken Institute School of Public Health, Washington, The George Washington University, DC. USA.
4. University of North Carolina at Chapel Hill, Gillings School of Global Public Health Sciences,Department of Epidemiology, North Carolina, USA.
5. National Institute of Environmental Health Sciences (NIEHS), Duram North Carolina, United States of America.
6. University of Ottawa, School of Epidemiology and Public Health (SEPH), Ottawa, Ontario Canada
7. School of Nutrition, Laval Université; Quebec, Quebec, Canada

Contents

Table S1: Epidemiological Studies of Vitamin D and Toxic Metals in Pregnant Women

eFigure 1: Vitamin D Status 25OHD

eFigure 2: Vitamin D Metabolism

eFigure 3: Serum Ca and P Balance

eFigure 4: Intestinal Absorption

eFigure 5: VDR/RXR and competition between EDC and 1,25OHD

Supplement Table S1: Epidemiological Studies of Vitamin D and Toxic Metals in Pregnant Women

**Reference**<br>**Study**<br>**Country**	**Sample Size, Recruitment Years**	**Exposure Type**	**Vitamin D measure**	**Concentrations**	**Results**	**Comments**
Arbuckle et al. 2016 [54]<br><br>MIREC Pregnancy Cohort<br><br>Canada	(n=1983)<br>Recruitment 2008-2011	Whole blood heavy metals (3rd trimester Cord blood) and Meconium	Vitamin D and Calcium intake (quartile) derived from FFQ given in the 2nd trimester of pregnancy	Geometric mean levels in 3rd trimester:<br><br>Pb: 0.57 µg/dL<br>Cd: 0.20 µg/L<br>Hg: 0.50 µg/L	Vitamin D intake negatively associated with 3rd trimester blood Cd, Pb and Mn and cord blood Pb.<br>Compared to Q4 of Vitamin D intake, Q1 had significantly higher 3rd trimester Cd concentrations	Bivariate descriptive analysis only.
Fisher et al. 2022 [55]<br><br>MIREC Pregnancy Cohort<br><br>Canada	(n=1983)<br><br>Recruitment 2008-2011	Whole blood Cadmium and Lead measured in the 1st, 3rd trimester	Serum 25-hydroxyvitamin D measured in the 1st, 3rd trimester	Geometric mean levels in 3rd trimester:<br>Pb: 0.57 µg/dL<br>Cd: 0.20 µg/L	Multivariate: bidirectional analysis using cross-lagged panel models:<br>1st trimester 25OHD was associated with 9% (-15%, -3%) lower 3rd trimester Cd and 3% (-7, 0.1%) lower Pb.	Covariates: smoking status, maternal age, education, ethnicity, fish consumption, household income, times spent outside, season
Fang et al. 2021 [56]<br><br>Prospective pregnancy cohort Hubei Province, China	(n=598 mother-newborn pairs)<br><br>Recruitment 2014-2015	20 urinary metals during pregnancy (1st, 2nd, 3rd trimester) including Pb, Cd, As,<br><br>Pb, Cd, Al, V, Cr, Mn, Co, Ni, Cu, Zn, As, Se, Rb, Sr, Ag, Cs, Ba, Tl, Th, U	Cord blood 25OHD		Percent differences in cord serum total 25OHD levels associated with each double increase in urinary metal concentrations during the whole pregnancy (single-metal model):<br><br>Cd: −6.74 (−13.90, 1.00)<br>As: 8.25 (0.77, 16.29)<br>Pb: −2.41 (−8.61, 4.22)	Covariates considered: maternal age, parity, gestational age, date of birth, mode of delivery, infant sex, socioeconomic level, lifestyle habits (smoking, alcohol consumption, supplements), prepregnancy BMI, gestational weight gain, season of birth

Reference Study Country	Sample Size, Recruitment Years	Exposure Type	Vitamin D measure	Concentrations	Results	Comments
Schell et al. 2003 [53]<br><br>Albany Pregnancy Infancy Lead Study (APILS) prospective pregnancy Cohort<br><br>United Staties	Recruitment 1986-1992; 1992-1998<br><br>(n=220 with two Pb measurement)	Whole blood Pb (1st, 2nd, 3rd trimester and delivery)<br><br>Cord blood Pb	National Cancer Institute Food Questionnaire: average nutrient intake across trimesters<br><br>Serum vitamin D (1,25OHD), 2nd and 3rd trimester	More than 50% of the women were below the RDA for zinc, calcium, iron, vitamin D and kilocalories.<br><br>Geometric mean maternal blood Pb concentrations ranged from 1.9 (1st trimester) to 2.2 (delivery). (µg/dL); Cord blood: 1.6 (µg/dL)	Higher maternal iron, calcium and vitamin D intakes related to lower cord blood lead levels.<br>A two SD reduction in vitamin D intake (10.5 to 2.4 mg) is associated with a 0.18 ug/dL increase in cord blood Pb.<br><br>No association with serum 1,25OHD and cord blood Pb.	Covariates: maternal age, education index, prepregnancy BMI, ethnicity, anthropometry, 2nd trimester Pb, delivery Pb, and kilocaloires
Jukic et al. 2021 [52]<br><br>LIFECODES pregnancy cohort – nested case-control study (preterm birth)<br><br>United States	Recruitment:2006-2008.<br><br>(n=381)<br><br>130 cases : singleton preterm births<br>352 unmatached non-preterm controls.	Urinary metals and elements (17) including As, Cd, Hg, Pb, measured at 26 weeks gestation	25-hydroxyvitamin D (25OHD) 10 weeks gestation	Geometric Mean concentrations of urinary metals (ppb) for those with low (<50ng/mL); high 25OHD (≥50 ng/mL) :<br>As: 19.2; 18.4<br>Hg: 0.54; 0.43<br>Pb: 0.33; 0.24	Low 25OHD (<50 nmol/L) was associated with a 47% increase in Pb levels. Low 25OHD vs High 25OHD ratio: 1.47 (1.07, 2.03).	Covariates: Specific gravity, age, race/ethnicity, education, body mass index, insurance type, gestational age, season.
Jukic et al. 2020 [57],<br><br>Maternal Vitamin D for Infant Growth (MDIG) trial<br><br>Intervention Study	Recruitment: March 2014-Sept 2015<br><br>(n=1300 pregnant women)	Whole Blood metals (Cd, Hg, Pb, Mn) measured in maternal and umbilical cord blood	Serum 250HD measured at baseline (17-24 wks gestation)<br><br>Participants randomized to receive weekly doses of:<br>• 4200 IU (n=118 maternal, 108 cord),	Median concentration:<br><br>Maternal at delivery (all participants)<br>Pb (µg/dL): 9.0<br>Cd (µg/L): 7.0<br>Hg (µg/L): 12.0	Unadjusted, intentional-to-treat linear regression<br><br>Maternal blood % change across supplement groups (4200, 16800 and 28000IU/wk) compared to placebo:<br>• Pb: 6.3% (-4.1, 18), 7.4% (-3.5, 20) and 6.0% (-3.4, 16)	Participants also offered 500 mg/d of calcium, 66 mg/d of elemental iron, and 350 ug/d of folic acid. Adherence high for Calcium (>85%).<br><br>Covariates for sensitivity analysis: gestational age

Reference<br>Study<br>Country	Sample Size,<br>Recruitment Years	Exposure Type	Vitamin D measure	Concentrations	Results	Comments
 Bangladesh		at time of delivery.	• 16800 IU (n=141 maternal, 104 cord) or<br>• 28000 IU (n=239 maternal, 216 cord) of vitamin D3 (cholecalciferol) or<br>• placebo throughout pregnancy.<br>n=no. of samples with blood metals measured.<br><br>Half of the women in the 28000 IU started doses prenatally and the other half postnatally.	Cord blood:<br>Pb (µg/dL): 7.6<br>Cd (µg/L):76% undetectable<br>Hg (µg/L): 22.0	• Cd: 6.6% (-5.0, 20), 4.9% (-7.0, 18), and 1.6 (-8.4, 13)<br>• Hg: -3.7% (-14, 8.0), 1.9 (-9.5, 15), -4.2 (-14, 6.2)<br><br>Cord blood % change across supplement groups (4200, 16800 and 28000IU/wk) compared to placebo:<br>• Pb: 8.5% (-3.5, 22**), 16% (3.3, 30**) and **11% (0.4, 23)**<br>• Cd: **2.2 (1.3, 3.7)**, 4.9 (-7.0, 18) and **1.7 (1.0, 2.9)** – due to low detection this is the relative risk of detection.<br>• Hg: 4.3% (-7.0, 17), 7.5% (-4.1, 20), and 6.3% (-3.9, 18)	at enrollment, treatment adherence, smoking status, anemia, BMI (12 mo. Postpartum), fish consumption, water source, asset index, month of enrollment.

eFigure 1:

Vitamin D Status 25OHD

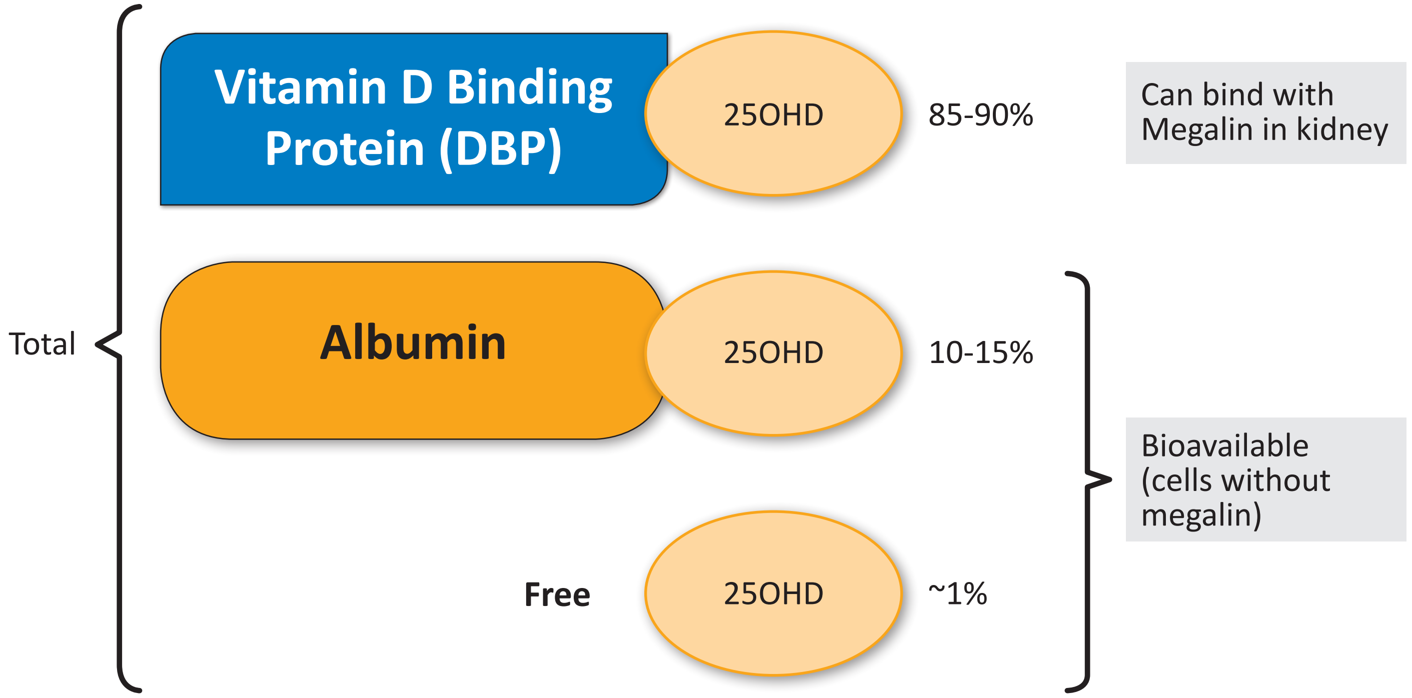

Adapted from Chun & Neilson. Vitamin D (4th ed). Vol 1: biochemistry, Physiology, and Diagnostics. Ch 51. 2018. pages 925-937;
Chun, R. F. et al. Vitamin D and DBP: The free hormone hypothesis revisited. J. Steroid Biochem. Mol. Biol. 144, 132–137 (2014).

- 25-hydroxyvitamin D: 25OHD
- Vitamin D Binding Protein (DBP)

The "free hormone" hypothesis proposes that only the non-bound portion ('free') of 1,25-dihydroxyviatmin D (1,25OHD) can enter cells and exert a biological effect. Nevertheless, in the proximal tubules of the kidney, megalin a multi-ligand receptor, acts as a cell-surface receptor for DBP so 25OHD bound to DBP can be internalized and subsequently hydroxylated to 1,25OHD. Although the action of megalin in the kidney is clear, its functional significance in a number of other vitamin D target tissues (placenta,mammary gland, parathyroid gland) are unknown.

eFigure 2:

Vitamin D metabolism

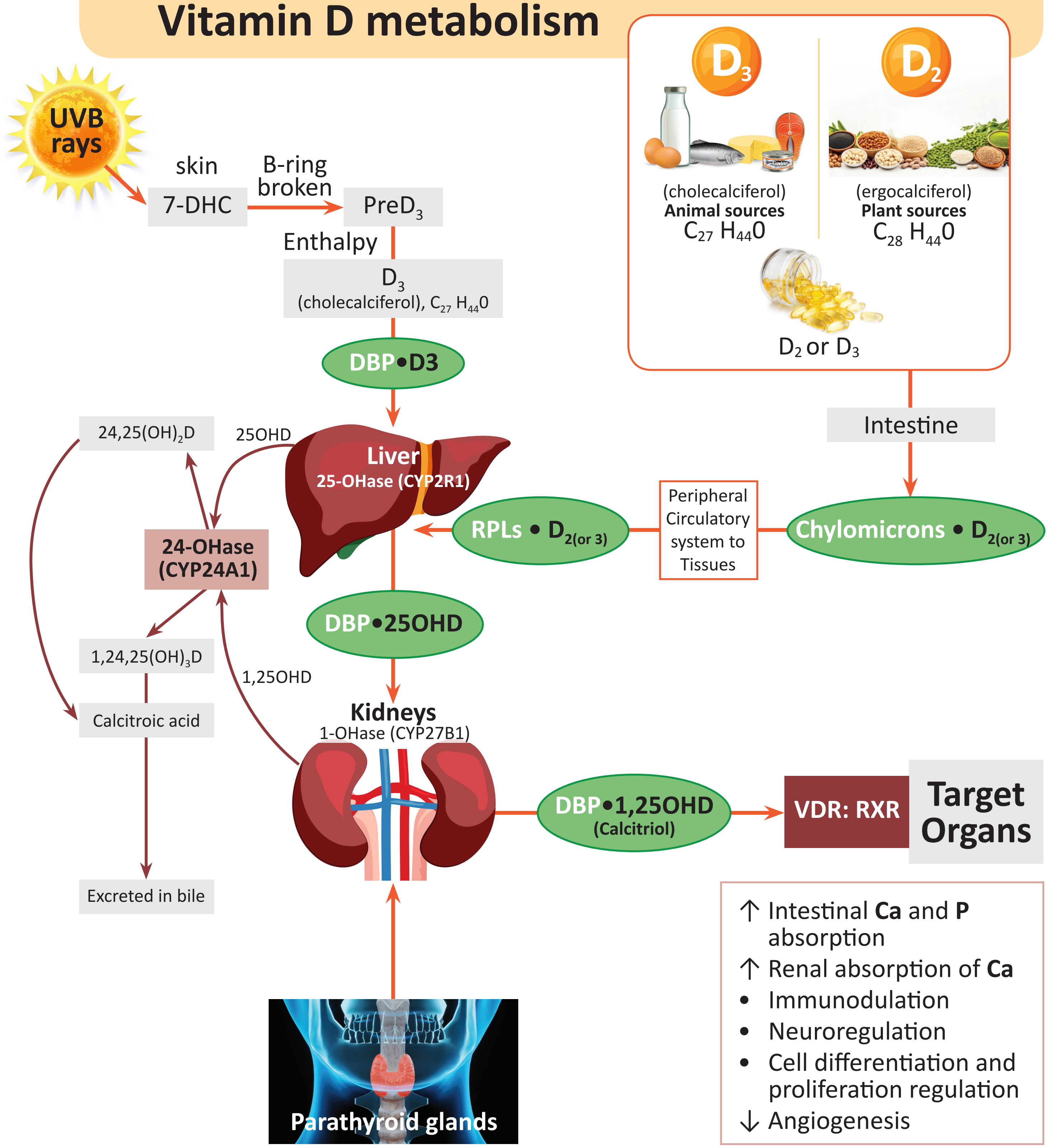

Modified from Holick (2009) and Mousavi et al. (2018)

Mousavi, S. E. et al. (2019) 'Air pollution, environmental chemicals, and smoking may trigger vitamin D deficiency: Evidence and potential mechanisms', Environment International. Elsevier, 122(September 2018), pp. 67–90.

Holick, M. F. (2009) 'Vitamin D Status: Measurement, Interpretation, and Clinical Application', Annals of Epidemiology, 19(2), pp. 73–78.

DBP: vitamin D binding protein; RPL: remnant lipoproteins

25-Ohase: 25-hydroxylase; 1-Ohase: 1α-hydroxylase; 24-Ohase: 24-hydroxylase

7-DHC: 7-dehydrochlesterol; VDR: vitamin D receptor; PTH: parathyroid Hormone;Ca: Calcium; P: Phosphate

25OHD: 25-hydroxyvitamin D; 1,25OHD: 1,25-dihydroxyvtitamin D

eFigure 3:

Serum Ca and P balance

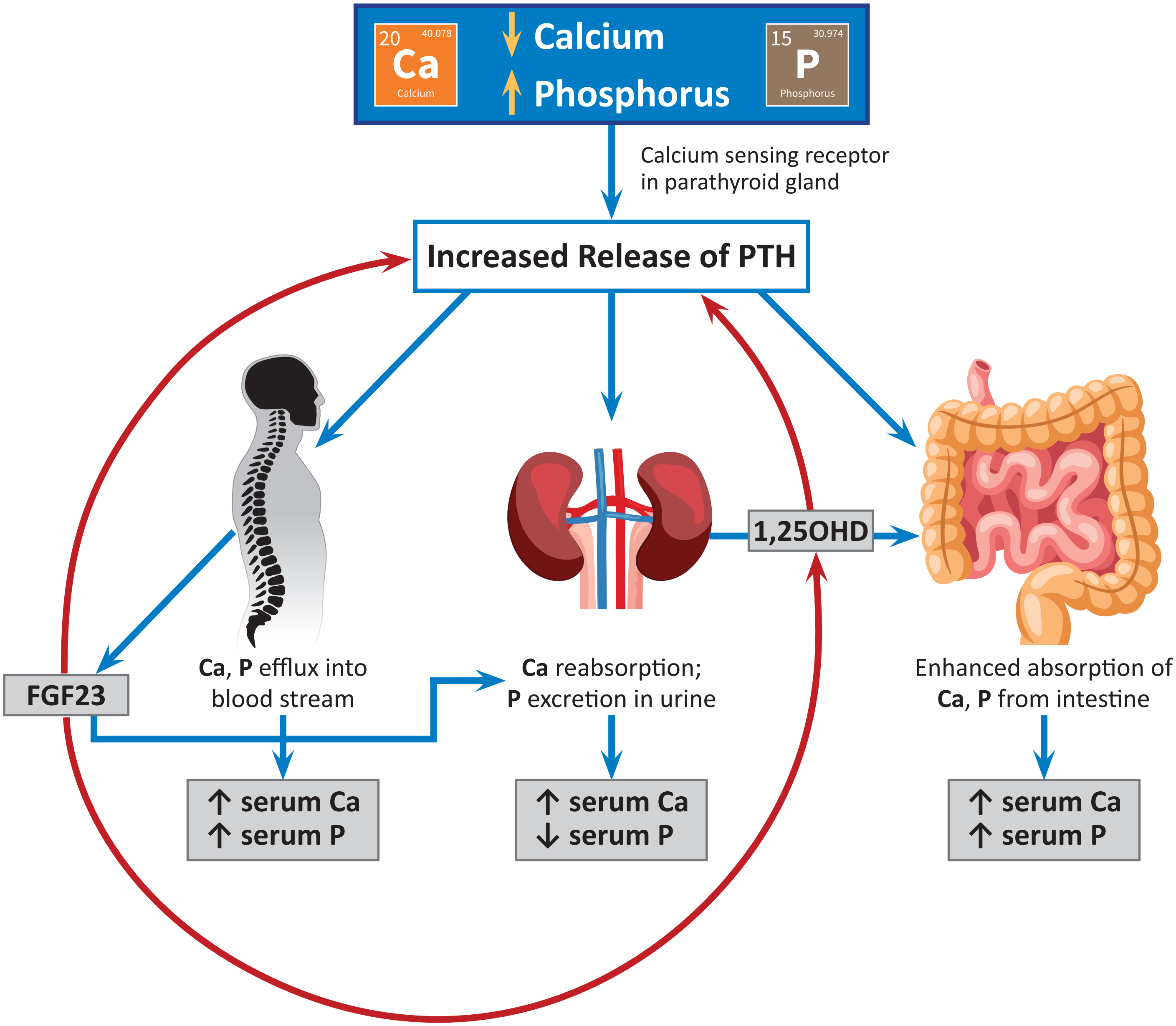

BLUE Means induces

RED Means inhibits

Adapted from: http://www.vivo.colostate.edu/hbooks/pathphys/endocrine/thyroid/pth.html

eFigure 4:
Intestinal Absorption

1-α-hydroxylase enzyme

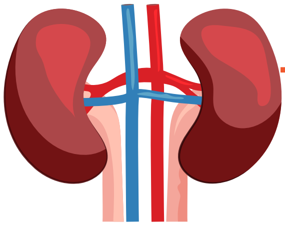

1,25OHD — Via DBP → **VDR** →

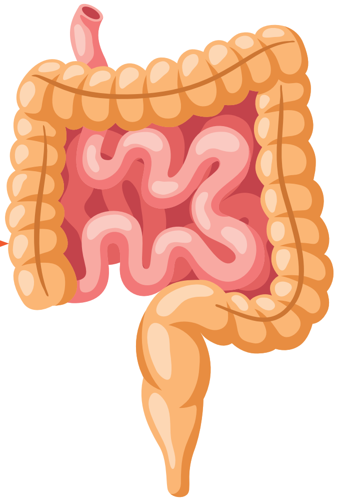

↓**Ca** and ↑PTH stimulates the production of 1,25OHD

↑ **Ca^{2+}-binding protein and** therefore increased intestinal absorption of **Ca**

The metals Pb^{2+} and Cd^{2+} are known to adversely influence renal production of 1,25OHD through the inhibition of **1-α-hydroxylase enzyme** in the renal tubules

The **Ca^{2+}-binding protein** in intestinal cells, responsible for Ca^{2+} absorption from the gut, also binds Pb^{2+} and Cd^{2+} and may be the reason for the absorption of these cations

Moon, J. (1994) 'The role of vitamin D in toxic metal absorption: A review', *Journal of the American College of Nutrition*, 13(6), pp. 559–564. doi: 10.1080/07315724. 1,25 dihydroxyvitamin D 1994.10718447. (1,25OHD) also known as calcitriol.

eFigure 5: VDR/RXR and competition between EDC and 1,25OHD

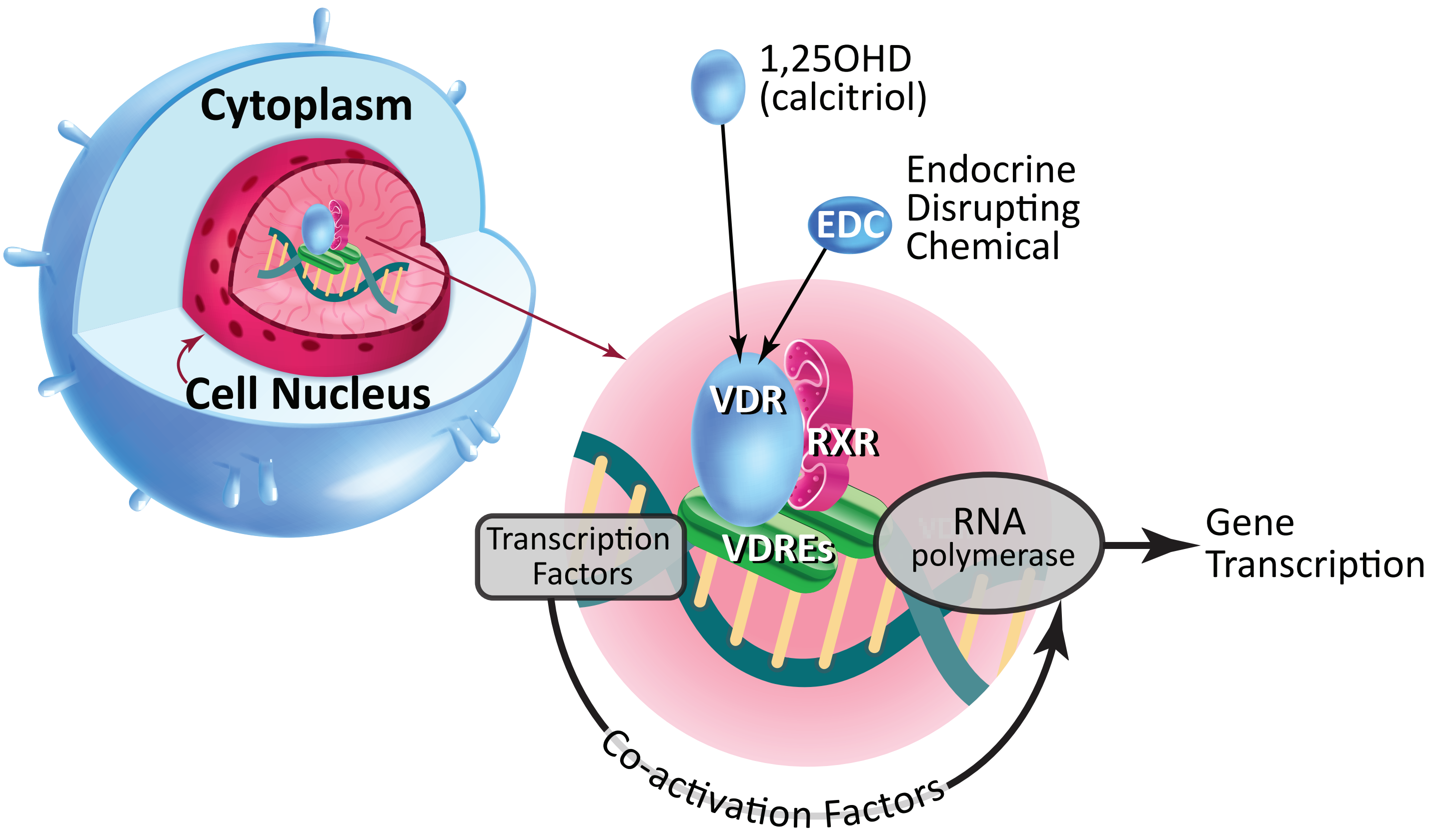

Endocrine Disrupting Chemicals (EDCs) have the ability to act as ligands and attach to specific hormone receptors (compete with calcitriol for VDR:RXR) which bind to response elements (VDREs) in target genes and regulating gene expression – thus producing downstream effects.

VDR: vitamin D receptor
RXR: retinoid X receptors
VDREs: vitamin D responsive elements
EDC: Endocrine Disrupting Chemical
1,25OHD: 1-25hydroxyvitamin D

Adapted from Raman, M. et al. (2011) ‘Vitamin D and gastrointestinal diseases: Inflammatory bowel disease and colorectal cancer’, Therapeutic Advances in Gastroenterology, 4(1), pp. 49–62. doi: 10.1177/1756283X10377820.
